# CoDA: Collaborative Data Aggregation in Emerging Sensor Networks Using Bio-Level Voronoi Diagrams

**DOI:** 10.3390/s16081235

**Published:** 2016-08-05

**Authors:** Chengpei Tang, Nian Yang

**Affiliations:** 1 School of Engineering, Sun Yat-Sen University, Guangzhou 510006, China; yangn8@mail2.sysu.edu.cn; 2 Research Institute of Sun Yat-Sen University in Shenzhen, Shenzhen 510006, China

**Keywords:** emerging sensor networks, collaborative data aggregation, Voronoi diagram, multi-hop wireless network, time synchronization

## Abstract

To implement minimum power consumption of the link, cluster heads adopt the multi-hop manner for inter-cluster communication so as to forward the aggregation data to the relay nodes. This paper proposes a collaborative data aggregation in emerging sensor networks using a bio-level Voronoi diagram, which is an energy-efficient data aggregation protocol that integrates topology control, Multiple Access Control (MAC) and routing. The sensor nodes situated in the lower level of the diagram are responsible for listening and gathering data, and should be organized by optimal clustering node. In the inter-cluster communication stage, a particle swarm optimization algorithm is addressed to seek optimal transmission path which could simultaneously achieve the minimization of the maximum next hop distance between two nodes in the routing path and the minimization of the maximum hop count, so the minimization of whole network energy consumption is realized. The results of theoretical analysis and simulation results show that energy efficiency and synchronization accuracy of the proposed algorithm can be much better than with traditional routing protocols, and the energy consumption of nodes in the whole network can be more balanced.

## 1. Introduction

With the development of the Internet of Things (IoT) industry, the application scope and complexity of emerging sensor networks (ESNs) have reached unprecedented heights. Against the background of the IoT, ESNs are highly dependent on a huge and complex network, which brings great reliability risks and presents many new problems and challenges to research on topology control in wireless sensor networks [1]. By deploying massive numbers of sensor nodes in the monitoring region, ESNs can collect the information of perceived objects effectively and deliver the fusion solution to the end user through wireless communication. Most applications of ESNs are focused on the field of military reconnaissance [2], environmental monitoring [3] and disaster relief [4], control and command in autonomous vehicles and robots [5], etc. However, the static sensor nodes are usually equipped with low processing and limited power capabilities, and often communicate over unreliable, short-range radio links. Since the communication range of sensor nodes is limited, they often are arranged in a self-organized manner and adopt hop-by-hop communication to exchange data. The prominent characteristics in the target monitoring applications of ESNs includes: many-to-one communication, the sensor nodes with greater density, and limited resources. Thus any communication protocol should strive for the timeliness and reliability of data transmission [5]. Among the factors that affect the performance of wireless sensor networks, topology control is one of the key problems [6]. If there is no topology control in wireless sensor networks, there will be the following problems [7]:

- Each node communicates with the maximum transmission power, and the limited node energy will be consumed quickly, thus reducing the lifetime of network.
- Large communication radius can increase the signal interference between nodes, which will affect the communication quality and reduce the network throughput.
- A lot of redundant edges exist in the generated network topology and result in network topology information redundancy. Large and complex routing calculation will bring about a waste of valuable computing resources.
- Non-uniform sparse link between nodes will impact on the network connectivity, reliability and survivability, especially in military application, which will directly affect the network security.

How to optimize the network topology, enhance the adaptability and robustness of the network topology, and provide a good topology support for the upper layer communication protocol is an important issue in the research of topology control and optimization. The existing literature shows that there are many constraints and challenges in the topology control of wireless sensor networks:

- Nodes’ dispersion [8]. In the face of different network applications, the nodes are distributed in two ways: predetermined and random dispersion. For the former, the data transmission can be carried out along a pre-designed path. For the latter, the distribution of nodes is not uniform and it is necessary to carry out reasonable clustering to ensure the network connectivity and energy efficiency.
- Energy consumption [9]. The lifetime of a network node depends on its battery life, and the communication and computation of the energy reserve form is very important. Because a node failure will change the topology, it may lead to the failure of the original algorithm.
- Heterogeneous nodes [10,11]. In many studies, it is usually assumed that the nodes are isomorphic, but in the face of different applications, the roles and capabilities of nodes may be different, which will lead to the emergence of many new problems in topology control.
- Tolerance [12]. The fault tolerance mechanism in the topology control is necessary, which can ensure the function of the network at the time some nodes out of action; for instance, lack of energy, physical damage or environmental interference.
- Scalability [13,14]. Large numbers of nodes are distributed in a sensing area, hence the design of a topology control scheme must be adapted to large-scale wireless sensor networks.
- Connectivity [15]. The connectivity of the network is constrained by the topology changes, node failure and random distribution of nodes.
- Coverage [16]. Due to the limitation of the communication radius of the sensor nodes, the area coverage is an important problem in the topology control of wireless sensor networks, especially for the mobile WSNs.
- Data fusion [17,18]. Considering the redundancy of the data collected by monitoring nodes, data fusion can be used to reduce the energy consumption by means of decease the packets being transmitted. Besides, it can also improve data accuracy. Thus the topology design should can provides an essential support for data fusion.
- Quality of service [19,20]. In some applications, the perception of the data should be issued within a certain time interval. Otherwise the data will be invalid. Especially for some important information, the reliability of data transmission is also very important, and the topology control algorithm should be designed to meet the requirement for different quality of service.

By using a reasonable topology control method, the system can work under a stable state for a long time and to improve the efficiency of data fusion. Therefore, it is needed to design a data aggregation protocol to integrate topology control, MAC and routing efficiently. Meanwhile, the communication path between neighbor nodes should be optimized to prolong the lifetime of network.

On the basis of existing research, a collaborative data aggregation in emerging sensor networks using a bio-level Voronoi diagram (CoDA) is proposed. The main idea is that all nodes in the network are distributed in the bio-level Voronoi diagram. The sensor nodes situated in the lower level of the diagram are responsible for listening and gathering data, and should be organized by some optimal clustering method. In each cluster, a cluster head is selected for gathering the data from its members and sending to the sink. In the upper level of the diagram, the relay nodes are deployed and constructed as the backbone network for forwarding data of all sensors to the sink node, in which the region covered by these nodes is relatively large and cluster heads adopt the manner of multi-hop for inter cluster communication so as to realize the integration of intra cluster data transmission to the backbone node. In the stage of inter cluster communication, a particle swarm optimization algorithm is addressed to seek optimal transmission path which could simultaneously reach the minimization of the maximum next hop distance between two nodes in the routing path and the minimization of the maximum hop count, so the minimization of whole network energy consumption was realized. Considering the time synchronization problem in multi-hop transmission mode, this paper proposes a multi-hop precision time synchronization model, which can achieve the balance between the synchronization accuracy and energy consumption.

The specific contributions of this paper include:

- A literature survey about various existing energy saving protocols and topology control approaches in ESNs, and analyze their advantages and disadvantages.
- An effective bio-level Voronoi diagram model based on Voronoi-cluster and relay nodes for ESNs is proposed.
- A collaborative data aggregation in emerging sensor networks using bio-level Voronoi diagram is proposed, which is an energy-efficient data aggregation protocol and integrates topology control, MAC and routing.
- Performance analysis of the proposed algorithm and an evaluation of the algorithm with respect to other traditional routing protocols.

The rest of this paper is outlined as follows. In Section 2 an overview of related works are presented. In Section 3, a collaborative data aggregation model using a bio-level Voronoi diagram is introduced with the presence of inhomogeneous time delays affecting the different hops. The multi-hop transmission mechanism and a multi-hop precision time synchronization model are presented in Section 4. In Section 5, the performance and features of the proposed algorithm are analyzed. Finally, conclusions are presented in Section 6.

## 2. Related Works

The research on ESNs includes many aspects, such as system survival time, routing selection and data fusion mechanism. Among them, how to reduce energy consumption is one of the key issues in ESN design, which directly determines the working lifespan of the network. Research shows that the communication between nodes is the main factor of the energy consumption in wireless sensor networks (WSNs), and some experts have put forward routing protocols with low energy consumption. The structure of self-organized sensor node clusters is proved to be a reasonable and effective mode to meet the need for sensors’ distributed deployment, which can save energy and optimize the topology of WSNs [21], however, the reasonable distribution of cluster heads can make the network energy consumption more stable and the energy distribution more uniform. In the early clustering protocol LEACH [22], it was proposed to select a node as cluster head in a rotation way for each node, so that the nodes can be elected as cluster heads as much as possible. This way makes the sensor nodes can be self-organized by an adaptive way. However, the problem is that the imbalance in energy consumption of the network is inevitable, and residual energy of the nodes is not taken into consideration of the cluster heads’ selection.

Subsequently, the cluster head selection algorithm is continuously optimized. In HEED [23], cluster head rotation and topology reconstruction were carried out in the whole network for a preset time period. In EDCR [24], Gamwarige et al. presented that the calculation of the energy threshold is adopted dynamically to trigger the cluster head rotation process. Although the rotation strategy can balance the network energy consumption, excessive alternation will influence the stabilization of the nodes’ organization and lead to heavy interruption of the transmission.

In [25], an improvement scheme was proposed based for traditional LEACH by using multiple conditions to determine the cluster head’s selection, which can reduce the energy consumption during the stage of cluster’s formation, and increase the efficiency of packet transmission. LEACH-V was proposed for resolving the problem of energy imbalance between the nodes and low utilization rate of the whole network [26]. The residual energy and the average energy of the network were considered during the process of calculating the threshold for cluster head’s selection. Also, a Voronoi diagram was used to restrict the situation of the cluster heads.

A two levels-weighted clustering algorithm (TL-WCA) was proposed for optimizing the routing path [27]. The network is divided into several clusters based on the improved competition mechanism. Then, the elected cluster head uses greedy algorithm to find the shortest path principle and then form a chain. Next, the nodes with more than the average energy in the chain and close to the sink are selected as the cluster head, and then execute the data fusion. In [28], the possible redundancy of cluster heads was analyzed, and a clustering routing algorithm based on cluster head redundancy was proposed to apply in industrial wireless sensor networks. When the energy of the cluster head was insufficient, the redundant cluster heads can be switched to the work state. For the isolated nodes that cannot communicate with any cluster head directly, the multi-hop routing mechanism based on a probabilistic model was used for data forwarding.

In [29], an analytical model for cluster head rotation was designed, which built a contention scheme for cluster head selection to remove the energy consumption in cluster head rotation. An EEHC algorithm for multi level energy heterogeneous network was proposed in [30], where the nodes with larger weights were more likely to become cluster heads. The main purpose of ESNs is that the sensors monitor the environment constantly and transmit the monitoring data to the cluster head, then forward the aggregation data to the sink through the cooperation of other nodes. From the point of view of data forwarding between nodes, multi-hop routing usually demonstrate more energy efficient than single hop routing, and it can avoid the energy-hole problem [31,32].

In [33], an equal cluster-based multi-hop routing (EMR) was proposed. The cluster head candidates for EMR were selected by their communication range and energy reserves. In addition, the communication between the cluster heads adopted a multi-hop manner and it is dominated by a weight indicating the energy-reserves and the distance among them. In [34], an energy-efficient clustering multi-hop routing (EECMHR) algorithm was proposed for resolving the problem of unbalanced energy consumption in traditional clustering routing. An average residual energy factor was introduced, i.e., the percentage of the cluster-head among other nodes was updated according to the ratio of each node’s residual energy and average residual energy within the cluster at the end of last round.

Wang et al. proposed an energy cost optimization model for the nodes deployed in Poisson distribution in wireless sensor networks [35]. Considering that multi-hop routing consumes less energy than single-hop routing, the relationships among the nodes’ density of Poisson distribution, optimal number of clusters and power consumption for multi-hop routing was examined. In [36], a clustering hierarchy arithmetic based on time delay and a multi-hop routing mechanism (CHTD-M) were presented. The algorithm makes the equally distributed cluster-heads of the network construct a routing tree, and it reduces the number of cluster head nodes connected directly with the base station in communication through the multi-hop transmission way.

## 3. System Model

Due to the diversity of the ESNs environment, topology control should be designed according to the characteristics of particular applications and take into account the state of sensor nodes. Also, there are some problems which affect the network performance, i.e., the network connectivity, coverage, reliability, robustness and fault-tolerant, as follows:

(1) The model is too ideal to consider many uncertain factors in practical application, which cannot meet the requirements of dynamic sensors distribution.
(2) Lack of effective measurement of the dynamic and self-adaptive network topology.
(3) Topology control mechanism or method should be designed in aspects of tolerance, high reliability and strong survivability.

This section will demonstrate a topology control model based on the Voronoi diagram, which can optimize the cluster structure and deploy the relay nodes reasonably.

### 3.1. Definition and Properties of Voronoi Cells

**Definition 1.**  *Given a set of n points* $S=\{p_{1},p_{2},\cdots ,p_{n}\}$ *in the field, the Voronoi diagram can be constructed by drawing the perpendicular bisector of line segment of each pair. Those bisector line segments form the boundaries of Voronoi cells are called Voronoi edges, and it can be defined as:*

(1) $$V(p_{i})=\underset{j\neq i}{\cap }\{p|d(p,p_{i})<d(p,p_{j})\},\;i=1,2,\cdots ,n$$

*where d(p,p_i_) denotes the Euclidean distance between points p and p_i_, and the area V(p_i_) is said to be the Voronoi cell of p_i_. Obviously, the Euclidian distance from the point in the region of V(p_i_) will be shorter than the points outside*.

Voronoi graphs have many interesting and surprising mathematical properties [37,38], such as influence region, lateral adjacency, liner behavior, largest empty circle, local dynamic characteristics, etc. These characteristics all provide a powerful tool to solve problems in spatial analysis, geography science, computer science and mathematics [39,40]. Among them, there are three main properties:

(1) Each Voronoi node is the intersection of the three Voronoi edges. If any node in the graph make a circle, which goes through the Voronoi edges corresponding to all the vertices (three or more), cannot incorporate any other vertex. The circle with the largest radius is called the maximum empty circle.
(2) For Voronoi polygons, Euler’s Regulation demonstrates that no more than six adjacent space targets can be influenced while a vertex is being deleted or added. This feature is consistent with the practical characteristics of node deployment and network topology in wireless sensor networks.
(3) For the points a,b∈V, the edge *ab* is a Delaunay edge if there is a circle through *a* and *b* so that all other points of *V* lie outside the circle. The collection of Delaunay edges defines a plane geometric graph *D*(*V*) known as the Delaunay triangulation of *V*. In the non-degenerate case, which excludes four or more points on a common circle, *D*(*V*) is indeed a triangulation. Even in degenerate cases, the faces of *D*(*V*) are convex polygons, and these can be further subdivided into triangles using additional edges.

### 3.2. The Model of Bio-Level Voronoi Diagram

**Figure 1:**
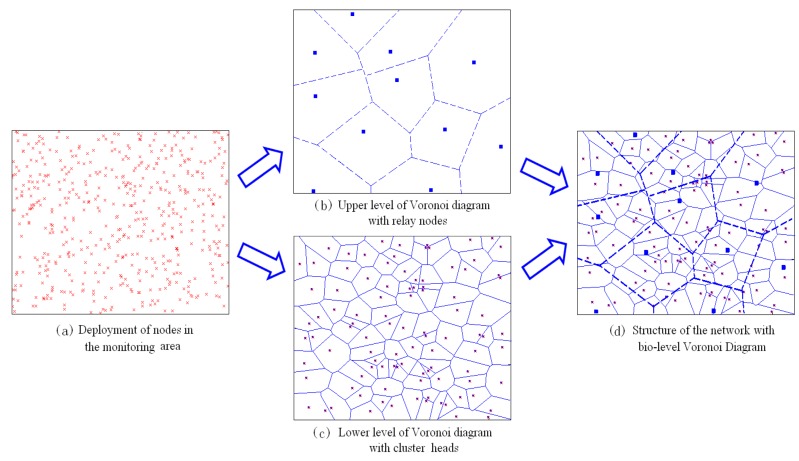
The model of a bio-level Voronoi diagram.

In the whole wireless sensor network, all the nodes are managed in the form of clusters, and the communication between the cluster head and its member nodes is carried out by single hop mode. The Voronoi diagram of the lower layer is composed of all the cluster heads, which reflects the adjacent distance and distribution of each cluster head. In addition, the upper Voronoi diagram is generated by the relay nodes, which constitute the backbone of the network. For each relay node, it owes a larger coverage range and can communication with various cluster heads, which attempt to transmit packets in multi-hop wireless routing. Therefore, the path optimization strategy should be designed for minimize the expense during the packets forwarding. Figure 1 show the bio-level Voronoi model presented in this paper. Especially, the division of the relay nodes in the upper Voronoi diagram is not absolute, and the cluster heads on the edge of the upper Voronoi diagram has the equal opportunity to select the appropriate relay node as the destination.

### 3.3. Structure of Voronoi-Cluster

From the point of view of the Voronoi structure corresponding to the computational geometry, the plane is divided into *N* zones by the Voronoi diagram, which depends on the number of discrete points *N*. The basic Voronoi diagram describes the areas that are nearest to a set of given points, and these can be viewed as zones of control [41]. Therefore, dividing the space plane into several regions and designating the sensor nodes into corresponding Voronoi-clusters, can ensure that all nodes in a single cluster are evenly distributed and make the distance between the member nodes to its cluster head as short as possible.

In ESNs, the selection of cluster heads plays a very important role in ensuring energy efficiency, and the main objective of cluster head selection is to make the cluster distribution reasonable. On the one hand, it is necessary to make the member nodes evenly distributed, so the communication overhead can be maintained at a low level. On the other hand, considering the inter-cluster communication, the distance between cluster head nodes should be within a reasonable range so as to keep the multi-hop transmission losses small. Therefore, to reduce the transmission losses and ensure insignificant difference between the member nodes in the aspect of cluster-in energy consumption, the topological structure of the network should be optimized. Meanwhile, by reducing the energy consumption in the multi-hop transmission as much as possible, ultimately it can achieve the balance of energy consumption in the whole network, and improve the life cycle of the network.

Due to the vulnerability of nodes and the variability of network topology, once some sensors with poor performance are selected as cluster heads it will seriously affect the quality of service of the network. The reliability of cluster heads and the distribution of clusters become the keys to determine whether the network can run efficiently.

In order to analyze the influence of the number of cluster heads on the overall energy consumption of the network, the first order radio model can be used for measuring energy consumption between sensor nodes. Equation (2) represents the amount of energy consumption in transmitting a packet with l bits over d distance according to the first order radio model [42]:

(2) $$E_{Tx}(l,d)=\left\{ \begin{array}{l} lE_{elec}+l\varepsilon_{fs}d^{2},d<d_{0}\\ lE_{elec}+l\varepsilon_{mp}d^{4},d\geq d_{0} \end{array}\right.$$

In which, $E_{elec}$ is the electronics energy, $\varepsilon_{mp}d^{4}$ is the amplifier energy of the next CH, and *d_0_* is a threshold. The energy consumption of *b* bits received by the receiver is $E_{RX}=lE_{elec}$.

The optimal number of clusters can be solved by calculating the energy consumed per round. It is assumed that the *N* sensor nodes are distributed evenly in the area of M×M, and the *k* cluster heads are generated by the election network. During the initial stage, the energy consumption depleted for message exchange between cluster head and its members can be calculated as:

(3) $$\begin{array}{l} E_{init}=kE_{CH-broad}+(N-k)E_{member-broad}\\ \;=k(l_{0}E_{elec}+l_{0}\varepsilon_{mp}R^{4})+(N-k)l_{0}E_{elec} \end{array}$$

In order to ensure that all nodes in the network receive the broadcast message, the radius *R* should be set as the maximum distance between any two nodes in the area. Under normal circumstances, $R>d_{0}$ and power amplifier consumption uses multipath fading channel model.

During the stage of the cluster formation, the *k* cluster heads receive the JOIN message sent by the respective member nodes and broadcasts the TDMA time slot table. Then, N−k member nodes send JOIN messages and receive the TDMA slot table. Because the distance between the cluster heads is relatively small, the power amplifier power consumption is free space channel model. The length of the JOIN message and the TDMA time slot table are set as $l_{JOIN}$ and $l_{TDMA}$ respectively, the energy consumption of the network can be calculated by:

(4) $$\begin{array}{l} E_{form}=k(E_{CH-JOIN}+E_{CH-TDMA})+(N-k)(E_{member-JOIN}+E_{member-TDMA})\\ =k[l_{JOIN}E_{elec}(\frac{N}{k}-1)]+l_{TDMA}E_{elec}+l_{TDMA}\varepsilon_{fs}d_{toCH}^{2}]+\\ \left(N-k)[l_{JOIN}E_{elec}+l_{TDMA}E_{elec}+l_{JOIN}\varepsilon_{fs}d_{toCH}^{2}\right] \end{array}$$

In the stable operation stage, *k* cluster heads receive the data collected by their member nodes respectively, and then send the aggregation resolution to the relay nodes through multi-hop. The transmission range in inter-cluster communication is often not more than, then the consumption of energy can be represented as:

(5) $$E_{cluster-in}=k[l_{0}E_{elec}+l_{0}\varepsilon_{mp}{\left(2*d_{toCH}\right)}^{2}]+kl_{0}E_{elec}$$

For the solution of $d_{\mathrm{toCH}}^{2}$ we can use the derivation in [43]:

(6) $$E[d_{toCH}^{2}]=\frac{M^{2}}{\pi k}$$

The total energy consumed in a round is:

(7) $$E_{total}=E_{init}+E_{form}+E_{cluster-in}$$

Let the partial derivative of $E_{total}$ to *k* is equal to zero, then the optimal number of clusters can be given as:

(8) $$k=\frac{N}{\sqrt{2\pi }}\times \sqrt{\frac{(2l_{TDMA}+l_{JOIN})\varepsilon_{fs}}{(N-l_{0})\varepsilon_{mp}}}$$

**Figure 2:**
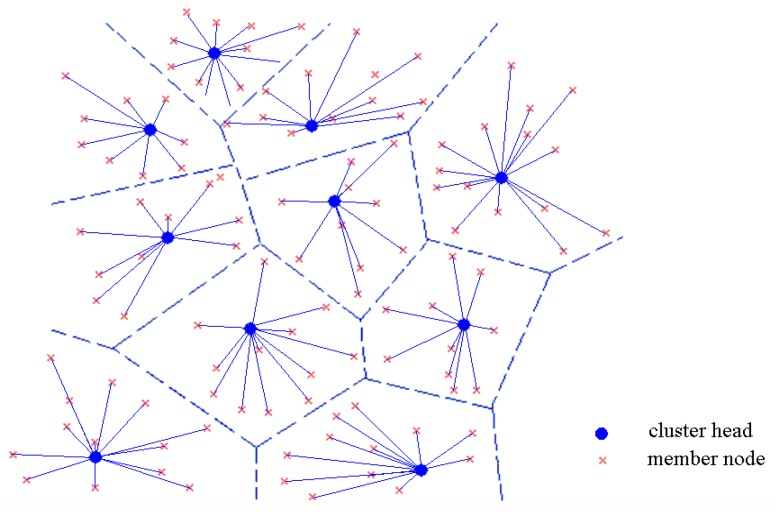
Dividing the monitoring area into Voronoi-clusters.

**Definition 2 (Voronoi-Cluster).**  *It is assumed that each node knows its own geographical position, and the network is divided into Voronoi cells. The sensor nodes situated in each Voronoi cell can be organized in a single cluster, and they may determine the corresponding Voronoi cell by the geographical position information. The structure of a Voronoi-cluster is shown as Figure 2*.

**Corollary 1.**  *In the lower level of Voronoi diagram, the distance between the cluster heads in different Voronoi-clusters has the same expected value*.

**Proof.**  Suppose that the Euclidean distance between node u and v is d(u,v) and the area of network is square of M×M. In order to ensure the full coverage of the network, the coverage radius of the cluster head can be defined as $\frac{M}{\sqrt{k\pi }}\leq r\leq \frac{2M}{\sqrt{k\pi }}$.

The distribution function of the node in the region can be expressed as:

(9) ζ(r)=p{d(u,v)≤r}

In Equation (9), the pairwise node of i and j is selected randomly, and the distance of other pairwise nodes in the network follows the same distribution. For a single cluster, there are N/k nodes in the wireless sensor network, and the number of pairwise nodes is $\frac{N}{k}*(\frac{N}{k}-1)/2$. Denote the distance of pair wise nodes is less than or equal to *r* as $X(\frac{N}{k},r)$, then $X(\frac{N}{k},r)$ is random variables and the expected value E{N(n,r)} can be expressed as:

(10) $$E\{X(\frac{N}{k},r)\}=\zeta (r)*\frac{N}{k}(\frac{N}{k}-1)/2$$

The probability function of the distance between nodes *u* and *v* limited by *r* is defined as:

(11) $$p((u,v),r)=\{\begin{array}{c} 1,d(u,v)\leq r\\ 0,d(u,b)>r \end{array}$$

Suppose $S_{N}^{i}$ is a sample of *N* nodes distributed in a wireless sensor network which are divided by Voronoi cells. For one node in a Voronoi region and $X(S_{N}^{i},r)$ denotes the number of nodes whose distance to other nodes is less than or equal to *r* in $S_{N}^{i}$, then:

(12) $$X(S_{N}^{i},r)=\sum_{i=1}^{N}\sum_{j=i+1}^{N}p((i,j),r)$$

Suppose that the probability for each candidate instance is $p(S_{N}^{i}$), then the expectation value $E=[X\left(S_{N}^{i},r\right)]$ is:

(13) $$E[X(S_{N}^{i},r)]=\sum_{i=1}^{K}\sum_{j=i+1}^{N}\left(X(S_{N}^{i},r)*p(S_{N}^{i})\right)$$

When *K* tends to infinity, if the total number of instances of the Voronoi cell in the wireless sensor network is brought into the formula, the expected value of the candidate cluster head distribution can be obtained. In the practical applications, it is very difficult to determine the value of *p*, which is related to the scale of the network and the density of nodes. When the cluster head selection is carried out in the Voronoi cell, the candidate cluster heads are subject to two-dimensional uniform distribution in the rectangular area. Because the selection of cluster head is taken place in a local area, it can ensure that the distribution of cluster heads is relatively uniform. □

**Definition 3.**  *Suppose* G=(V,E)*, and G being called as a simple undirected graph should satisfy the following conditions:*

*(1)*  G is a connected undirected graph without a self-circle.
*(2)*  There is at least one side between any two nodes in G.

**Figure 3:**
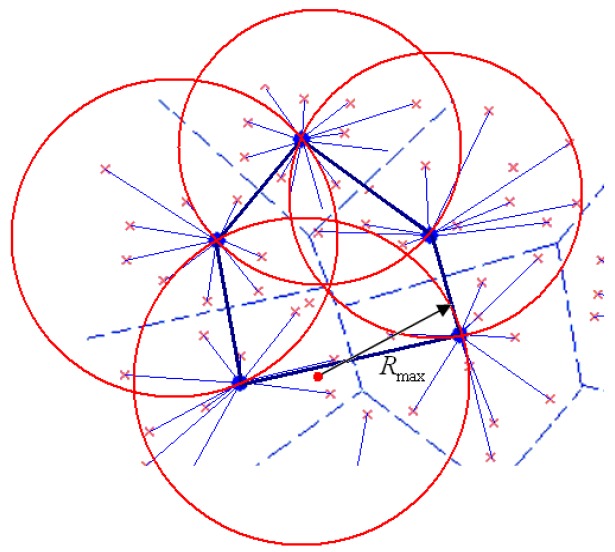
The minimum communication radius for inter-cluster transmission.

Graph *G* should be guaranteed with k-coverage and k-connectivity, and the single hop communication mode is adopted for cluster-in communication. In the existing clustering schemes, the nodes in the cluster can choose multi-hop or single-hop mode to transmit their gathering data to the cluster head. In order to balance the energy of the cluster head nodes often need to be replaced regularly, and the members may also change. If using multi-hop communication, each rotation for cluster head selection need rebuild the routing tree, which will result in high overhead and hard to maintain. Figure 3 shows the minimum communication radius for inter-cluster transmission.

To determine the minimum radius of inter-cluster transmission, the proposed Algorithm 1 is designed as following: **Algorithm 1.** Determination of the minimum transmission radius for inters cluster communication**.**Input: G=(V,E), Vertex set of V(S)Output: minimum communication radius $R_{\max}$1. $R_{\max}=0$;2.  Create the Voronoi diagram V (S) of the point set;3.  for each node i∈S do4.    Calculate Convex Hull $CH(S_{i})$;5. end for;6. for each v∈V(S)7.  if v is located inside the rectangle, then8.        Calculate the radius of circle with v as the center;9.        $R_{\max}=R_{v}$;10. end for;11. for each edge e∈CH(S)12.    Find the point x of perpendicular and rectangular of e;13.    Calculate the distance dist(x,e) between x and the endpoints of e;14.    if $dist(x,e)>R_{\max}$ then15.        $R_{\max}=dist(x,e)$;16.    end if;17. end for;

### 3.4. Formation of the Voronoi-Cluster

During the process of cluster formation, each cluster head records the information near the other cluster heads, including the remaining energy and the distance between nodes. The specific steps are described as follows:

Step 1: There are *N* sensor nodes and a sink node which are deployed on 2-D plane, and *k* cluster heads are selected by sink according to the residual energy and geographical position;
Step 2: The monitoring area is divided into *k* Voronoi cells in terms of the cluster heads’ position;
Step 3: Cluster head *CH_i_* broadcasts the message, including its residual energy and ID, to adjacent nodes. The other cluster heads which can receive the information will restore to the memory and generate their own node list, including the residual energy of the source node, and the distance between them.
Step 4: Next, find out the center node among all cluster heads in a single Voronoi cell. The center node sends *Center Declare Message* (CDM) to other cluster heads, and adds the nodes to the set by $SendMessage(ID_{u},CDM)$.
Step 5: After working for some time, if ResEnergy(u)<ResEnergy(v),$v\in nodelist_{u}$, node *u* will give up the role of center node and notify all cluster heads of the set.
Step 6: Calculate the distance between all adjacent cluster heads. If $\min\{d(u,v)\}<d_{0}$, combine the Voronoi cells with adjacent cluster heads and constitute the links for all member nodes.
Step 7: All cluster heads send *Active Dynamic Information* (ADI) message to others periodically. Once a cluster head failures, other cluster heads can perceive quickly and then output the backup set. The partition of the link caused by cluster head failure can be fixed as far as possible, so as to reduce the loss of data and ensure the reliability of transmission.

### 3.5. Deployment of Relay Nodes

The research shows that the use of the multi-hop communication manner between sensor nodes can improve the energy efficiency and prolong the network lifetime, but it also brings some problems, for example, more delay for packet forwarding, and the formation of energy holes owing to the excessive overhead for the nodes close to the sink.

Therefore, the data aggregation mechanism proposed in this paper divides the nodes of the whole network into three levels, consisting of the cluster head, the member node and the relay nodes. The backbone network topology is formed by arranging a small amount of powerful relay nodes in the network, which can achieve inter cluster communication efficiently and reduce the delay of the data transmitted from the monitoring sensor to the sink effectively. Relay nodes usually are equipped with strong energy supply, communication capacity and computing power. In contrast with cluster heads, they possess much wider coverage and are responsible for data aggregation from adjacent clusters. Obviously, the bio-level structure of the node is adaptive for nodes’ management. Meanwhile, it has a good scalability.

For the lower level network, each cluster head can communicate with corresponding relay node by using multi hop mode. In the upper level topology, relay nodes consist of the backbone network. In order to reduce the communication cost, the number of relay nodes should be as less as possible under the premise of connectivity.

**Definition 4.**  *If* $s_{j}$ *satisfies the condition:* $N_{c}(i)=\{s_{i}|d(s_{i},s_{j})\leq R_{c},j\neq i\}$*, then* $s_{j}$ *is the neighbor node of* $s_{i}$*, where* $R_{c}$ *denotes the transmission radius and* $s_{j}$ *is the neighbor node of* $s_{i}$.

**Definition 5.**  *If any point in a region is within the scope of coverage from any node in the set* S*, and satisfy that the points in S are connected, then,* S *can be defined as a connected coverage set*.

**Definition 6.**  *If the minimal nodes can constitute a connected coverage set, then the set* $S=\{s_{1},s_{2},\cdots ,s_{n}\}$ *can be defined as a minimum connected coverage set*.

To determine the minimum communication radius of inters cluster communication, the proposed Algorithm 2 is designed as following: **Algorithm 2.** Determination of the minimum communication radius of inters cluster communication.Input: the sensor node set $S=\{s_{1},s_{2},\cdots ,s_{N}\}$, the number of cluster head *k.*Output: the set of relay nodes *RS.*1. Suppose all nodes in the set S as generic point, generates the corresponding k order Voronoi diagram $V_{k}(S)$;2. The set of polygon for Voronoi diagram is denoted as $V=\{V_{k}(PL_{1},S),$
$V_{k}(PL_{2},S),$
$\cdots ,V_{k}(PL_{M},S)\}$, where $V_{k}(PL_{1},S)$ is the i-th polygon for $V_{k}(S)$;3. RS=∅;4. while V≠∅ do5.    for each $V_{k}(PL_{i},S)\in V$ do6.      if $s_{j}$ is the generic point of polygon $V_{k}(PL_{i},S)$ then7.        record the occurrence time of node $s_{j}$;8.      end if;9.   end for;10. Find out the $s_{\max}$ which denotes the maximum number of occurrences of generic point;11. The node $s_{\max}$ can be added in the set of RS;12. $V=V-\{V_{k}(PL_{i},S)|s_{\max}\in PL_{i}\}$;13. end while.

## 4. Multi-Hop Transmission and Synchronization

Since the energy consumption of data transmission increases exponentially with the distance between nodes, it is necessary to form a backbone network among the nodes in the cluster head and the hot spot area to realize the multi-hop communication so as to save energy consumption. In this paper, a particle swarm optimization algorithm is designed, which makes use of a multi-objective adaptive value function to find the optimal routing tree and satisfy the minimum energy consumption condition.

### 4.1. Fitness Function

In the case of the particle swarm optimization algorithm applied to the problem domain, the set of fitness functions is directly determined by the selection and removal of the transmission path. In order to minimize the energy consumption of the whole network, the design of the fitness function should satisfy the following two conditions: (1) The distance between the nodes of any hop should be as small as possible; (2) The maximum number of hops in the monitored area should be as small as possible. Therefore, the fitness function can be constructed as:

(14) $$\left\{ \begin{array}{l} fitness=w_{1}*MaxDist+w_{2}*MaxHops+w_{3}*Direc\\ 0\leq w_{i}\leq 1,\;\sum w_{i}=1 \end{array}\right.$$

where $w_{1}$, $w_{2}$ and $w_{3}$ are the distance factor, the hop factor and the direction factor, respectively. MaxDist is the maximum communication distance between any adjacent two-hop nodes in the monitoring area, MaxHops is the maximum number of hops in all transmission paths, and Direc denotes the deviation angle between the source node and the relay node.

### 4.2. Initialization of Particle Swarm Optimization 

The particle is defined as the different paths of the data transmission, and each particle’s dimension M is the same, which is corresponding to the number of cluster heads in a cell in the upper Voronoi diagram. The position of particle i is initialized of Pos(i,σ) randomly, Pos(i,σ)∈Rand[0,1] and 1≤σ≤M. The method of selecting the next hop node $S_{d}$ for node $S_{S}$ is as follows:

(15) $$s_{d}=Index(NextHop(s_{s}),n)$$

where $Index(NextHop(S_{S}),n)$ is the index function of returning the value of the *n*th cell in the set $NextHop(S_{S})$. $NextHop(S_{S})$ denotes the candidate nodes for the next-hop, and the value of n is determined by the formula:

(16) $$n=\left\lceil Pos(i,\sigma )\times |NextHop(s_{s})\right\rceil$$

In the phase of data communication, the specific steps of using particle swarm optimization algorithm to select the optimal path are as follows:

Step 1: Initialize the particle *i* per dimension with random velocity Pos(i,σ);
Step 2: Mapping particle *i* routing tree;
Step 3: According to the routing tree can obtain MaxDist and MaxHops;
Step 4: Calculate the fitness value fitness(Pos(i,σ) of each particle;
Step 5: Finding the individual extreme value $P_{best\_i}$ and the global extreme value $Global_{best}$;
Step 6: Update the speed and position of the particles and make the corresponding adjustment;
Step 7: Repeat Steps 2 to 6 until the threshold number of iterations;
Step 8: According to the individual extreme value and the global extreme value in Step 5, the optimal path from node *i* to the relay node can be determined.

### 4.3. Precision Time Synchronization Model

In distributed systems, different nodes have their own local clock. Due to the frequency of different nodes of the crystal oscillator frequency deviation, as well as temperature changes and electromagnetic interference and so on, their time clock will gradually deviats even if at a certain moment all nodes can achieve time synchronization. In the multi-hop mode, time synchronization issues need more attention in order to ensure that the data collected by different clusters can be accurately reached by the relay nodes.

**Figure 4:**
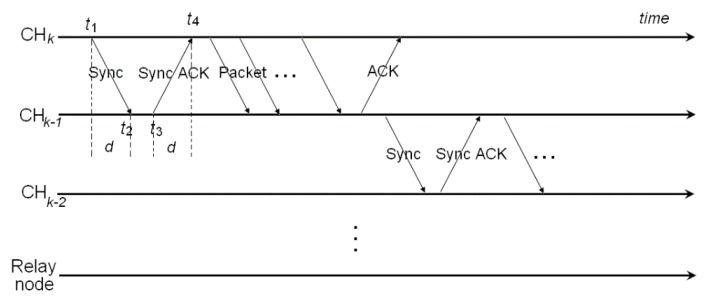
Time stamp exchange of PTP in multi-hop mode.

The precision clock synchronization protocol (PTP) is a high precision time synchronization protocol which is applied in industrial measurement and control system. In the process of network communication, its synchronization accuracy can still be controlled below the subtle level, which can effectively meet the requirements of real-time performance in distributed systems. In this paper, the basic mechanism of multi-hop time synchronization is designed based on the PTP protocol idea, and the time stamp exchange of PTP protocol in multi-hop mode is shown in Figure 4.

The horizontal axis represents the time interval, and demonstrates the process while the cluster head nodes forward data packets to a relay node in single hop or multi-hop manner. For the time synchronization in the first hop, $CH_{k-1}$ is the slave node of $CH_{k}$. However, the role of $CH_{k-1}$ will become the master node for $CH_{k-2}$, and so on. Actually, there exists a clock offset between the master and slave nodes, and time delay happens in the process of packet switching. If the slave nodes and the master node can calculate the clock offset by exchanging time stamps, the time synchronization can be achieved.

The basic principle is that the slave node can calculate the clock offset θ through the exchange message from the master node, which contains the time stamp information, then estimate the actual time of the master node and adjust the local clock. The time stamp exchange process of PTP protocol is shown in Figure 4. As can be seen from the Figure 4, $t_{1}$ and $t_{2}$ are the time stamps of a master node and slave node during an interaction for time synchronization respectively. $t_{3}$ and $t_{4}$ represent the time stamps of a master node and slave node derived from the delay request message in the following process. Considering the asymmetry of the transmission delay between the master and slave nodes, a linear equation set is established for the PTP synchronous packet switching process:

(17) $$\left\{ \begin{array}{l} t_{2}=t_{1}+\theta +d_{ms}\\ t_{4}=t_{3}-\theta +d_{sm} \end{array}\right.$$

Then:

(18) $$\theta =\frac{\left(t_{2}-t_{1})-(t_{4}-t_{3}\right)}{2}+\frac{d_{sm}-d_{ms}}{2}$$

Similarly, time synchronization in multiple rounds can be derived from the above function. While the time synchronization happens at the round t, the clock offset between the master node and slave node can be expressed as:

(19) $$\theta_{t}=\frac{1}{t}\sum_{i=1}^{t}\frac{\left[t_{2}^{\left(i\right)}-t_{1}^{\left(i\right)}]-[t_{4}^{\left(i\right)}-t_{3}^{\left(i\right)}\right]}{2}+\Delta d$$

where $\Delta d=\frac{d_{sm}-d_{ms}}{2}$ is a Gauss random variable with mean value of 0 and the variance of $\sigma_{d}^{2}/2$.

In the process of synchronous packet transmission, the discrete clock model is adopted to resolve the optimization problem in the case of the noise being doped [44,45]. Then, the time deviation of Receiver-Only synchronization mode can be given as:

(20) $$\left\{ \begin{array}{l} \theta (t)=\theta (CH_{k},\Delta t)-\theta (CH_{k-1},\Delta t)+\alpha [t]\tau [t]+v[\Delta t]\\ \Delta (t)=\{[\theta (CH_{k},t)-\theta (CH_{k},t-1)],[\theta (CH_{k-1},t)-\theta (CH_{k-1},t-1)]\} \end{array}\right.$$

Once monitoring the SYNC message, the slave node begins to calculate the length of the synchronization beacon, count the number of synchronized packets received and the measure the transmission delay. Furthermore, it can track the unstable clock drift of the master node to maintain the minimum offset state by Calman information gain. When the observation noise is $\sigma_{v}^{2}$, the gain formula is as follows:

(21) $$GF(CH_{k},CH_{k-1})=\sqrt{\frac{\sum SYNC(CH_{k},CH_{k-1})\times \theta (t)}{\sigma_{v}^{2}+\sum SYNC(CH_{k},CH_{k-1})\times \theta^{2}(t)}}$$

where $SYNC(CH_{k},CH_{k-1})$ denotes the length of the synchronization beacon between $CH_{k}$ and $CH_{k-1}$ in every time of synchronization.

When the synchronous data packet transmission is finished, the clock drift can be obtained by the formula:

(22) $$\hat{\theta }[t]=\hat{\theta }[t-1]+GF(CH_{k},CH_{k-1})\times \theta [\Delta t]$$

Therefore, the clock offset of the node can be tracked in a timely manner, and the synchronization error can be accurately calculated to obtain a relatively small clock offset. 

## 5. Simulation Results

This study uses a simulation for performance evaluation of the proposed protocol, which uses the network simulation software NS2 [46] to set up the model and environment needed by the experiment. In the experiment, we assume that the sensor nodes are distributed in a square area, and the location of each sensor node is randomly generated. In order to reduce the error, we repeat the experiment 100 times.

**Figure 5:**
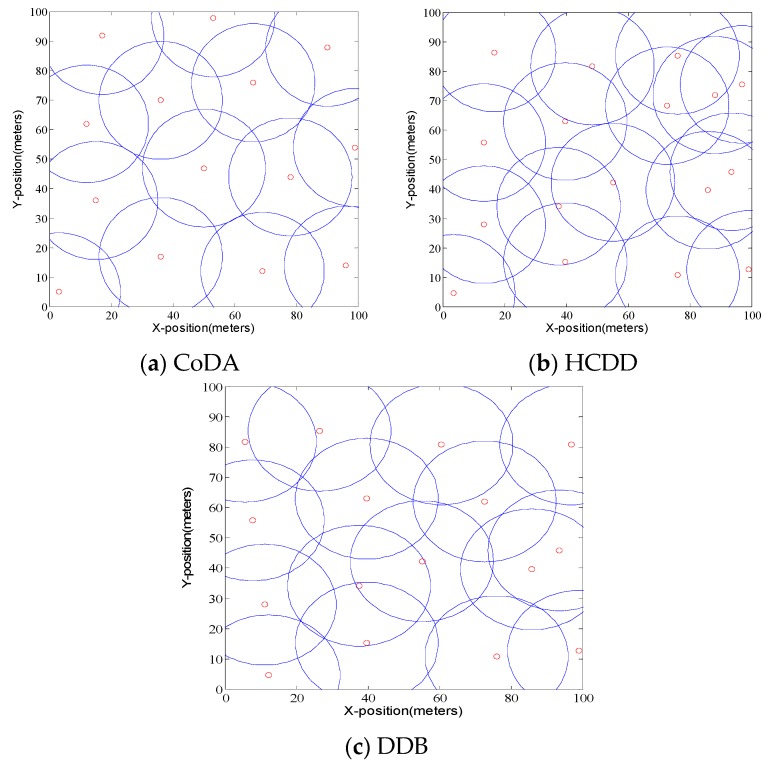
The placement of relay nodes in different protocols.

Figure 5 shows the placement of relay nodes and their coverage area in CoDA, HCDD [47], and DDB [48], where there are 200 sensor nodes distributed in the field of four square sides with a length of 400 m.

**Figure 6:**
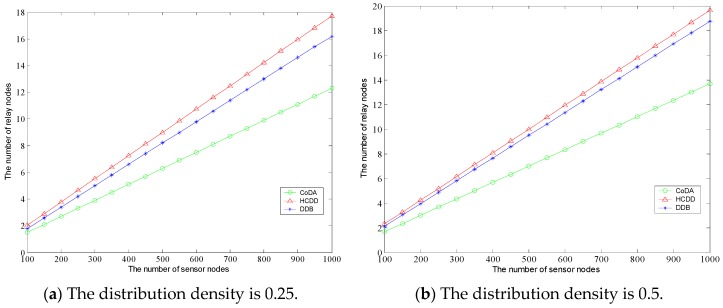
Comparison of number of relay nodes in different distribution density.

By comparison, we can find that the number of relay nodes in CoDA is 14, which is a minimum feasible topology for relay nodes. Also, HCDD and DDB can achieve the coverage of the whole network by deploying more relay nodes, but the number of relay nodes is 16 and 17, respectively. Next, the number of relay nodes required by each algorithm is analyzed under different experimental scenarios. Two sets of experiments are carried out, where the communication radius of the sensor nodes is set to 50 m, and the communication radius of the relay node is 100 m. The number of sensor nodes deployed in the network increased from 200 to 800. The tests were repeated 10 times, respectively, and the average number of required relay nodes was obtained. Figure 6a,b show the number of relay nodes required for each algorithm with different node density, where the horizontal axis represents the number of sensor nodes and the vertical axis represents the number of relay nodes. In the first scenario of experiments, the density of the sensor is set to 0.25, and the density of the sensor is 0.5 in the second scenario. It can be seen that the number of relay nodes for each algorithm has a linear increasing trend under the condition that the distribution density of sensor nodes is constant.

**Figure 7:**
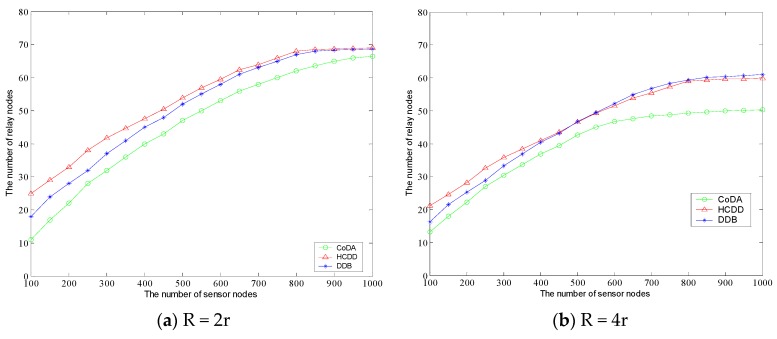
Comparison of number of relay nodes in different coverage radius.

As it can be seen from the Figure 6, the number of relay nodes demonstrates a linear increasing trend under the condition that the distribution density of sensor nodes being kept constantly. When the number of sensor nodes is equal, the number of relay nodes in HCDD is of the most, and the least number of relay nodes in CoDA can cover most of the area. According to the slope of the graph, with the increase of the number of sensor nodes, the gap between the numbers of relay nodes being arranged in the three algorithms is more and more large. By contrast between Figure 6a and 6b, we can observer that the greater the distribution density is, the fewer the number of relay nodes need to be arranged, which demonstrates the characteristic of geometric coverage for the relay node arrangement. Next, the number of relay nodes generated by different protocols is analyzed under the condition that the region size is fixed and the radius of the relay node is different. In two scenarios, the sensor nodes are evenly distributed in the 800 m × 800 m fixed area. The sensor node communication radius r = 50 m, the communication radius of relay node is 100 m and 200 m. As shown in Figure 7a,b, with the increase of the density of sensor nodes, the number of nodes generated is gradually increased, and the rate of growth slows down gradually in the fixed area. It can be observed that the distribution density is sensitive to the variation of *R*.

Coverage rate is usually regarded as an index for measuring the quality of service [49,50]. Here, We define it as a proportion of active nodes in the network at a certain time t, i.e., $Cov(t)=1-\frac{n_{b}(t)}{N}$, where $n_{b}(t)$ is the number of nodes out of range of arbitrary relay nodes. In this test, the transmission radius of all relay nodes is fixed so as to analyze the relationship between the coverage rate and the number of relay nodes in different protocols. 

**Figure 8:**
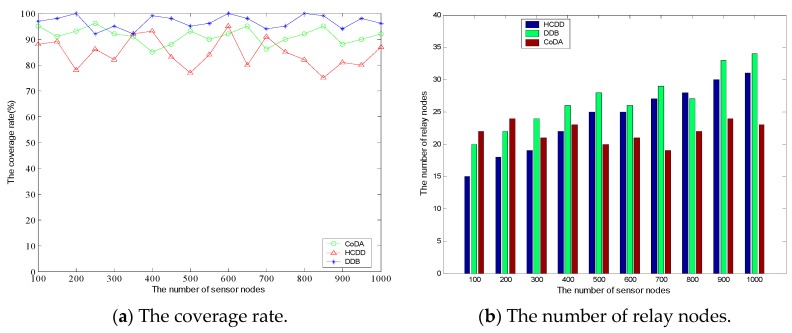
Comparison of relationship between the coverage rate and the number of relay nodes.

Figure 8a shows the variation of coverage rate. It can be seen that the stability of DBB and CoDA is relatively better than HCDD, and they can maintain a high coverage rate. When the number of nodes is small, the coverage rate of HCDD can be maintained at a stable level. However, with the increase of the number of nodes, the coverage rate fluctuates greatly, which shows that the algorithm is greatly affected by the node’s transmission radius. Figure 8b shows the corresponding number of relay nodes. We can find that the deployment of relay nodes increases rapidly and thus achieves a higher coverage rate in DDB while the node density increases. In other words, the coverage rate of DDB is implemented by generating more relay nodes. It will cause too much redundancy of relay nodes and may result in serious communication interference. The number of relay nodes in CoDA is relatively stable at this point and indicates the reasonable deployment of relay nodes and topology control can realize the high coverage.

**Figure 9:**
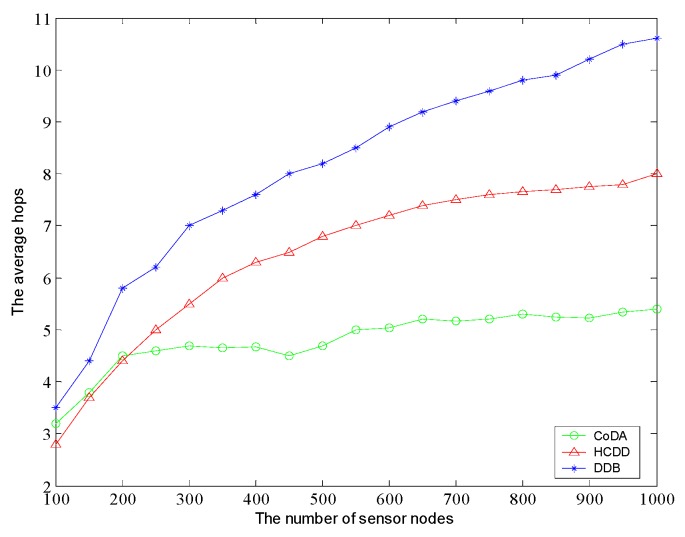
Comparison of average path length.

Further, we analyze the data collecting path length. Because those protocols are all applied with multi-hop transmission, we do not constrain the communication radius of relay nodes in the experiments. Then, the communication radius are adaptively adjusted in each protocol using the most energy-efficient way, and average hop count is statistically evaluated. As shown in Figure 9, we can see the increase of the number of nodes, the hop corresponding growth trends. While the node number reaches 1000, the path hops of HCDD has increased more than 10. As a consequence, this inevitably leads to high network latency and it can’t be better adaptive to the delay-sensitive applications with HCDD protocol. On the other hand, the basic hops of CoDA protocol for data acquisition can always keep within five hops. In conclusion, the cluster head can forward packets to the relay node via other cluster heads and this reduces the number of forwarding steps, which may benefit from the relay nodes of reasonable deployment, reasonable planning for the lower level Voronoi diagram, and the application of particle swarm optimization.

**Figure 10:**
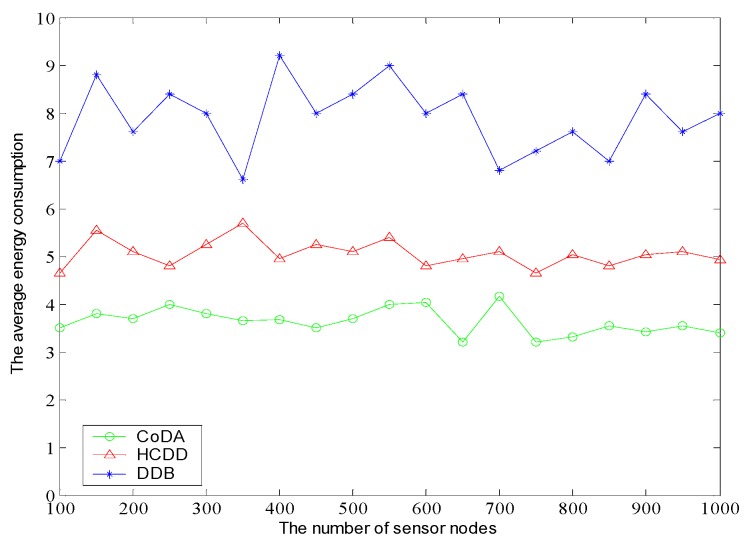
Comparison of average energy consumption of sensor nodes.

In order to analyze the equilibrium characteristics of the node average energy consumption, we deploy 200 nodes with unique identification numbers and analyze the average energy consumption of these nodes in 100 rounds [51,52]. The average energy consumption of nodes with different number of sensors is shown in Figure 10. From the result, we can see that the node’s energy consumption of CoDA is more balanced rather than the HCDD and DDB. This is because the CoDA can manage the nodes hierarchically via double Voronoi diagram. Meanwhile, the CoDA can determine the cluster heads via the optimal clustering method. As a result, the node average energy consumption remains at a lower level. At the same time, the cluster heads can also balance well the energy consumption of different cluster heads in multi-hop transmission mode through the optimized path forwarding to relay nodes, which can avoid the overloaded energy consumption. 

**Figure 11:**
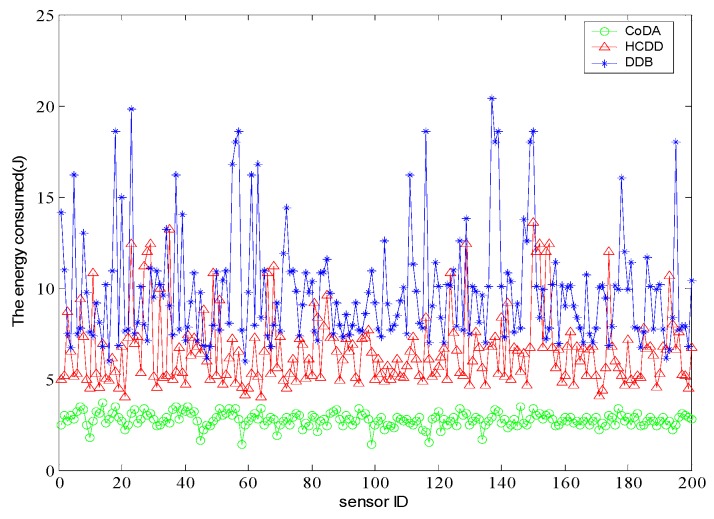
Comparison of energy consumed by each node.

The energy consumption of the each node is shown in Figure 11, and the energy consumption of the top 20 clusters is more comparable to the latter. The energy consumption in HCDD and CoDA is more smooth and lower than in DDB. The DDB protocol adopts the polling mode method to select cluster heads with same probability, and there may be a chance that a cluster head will die earlier and cause the hot-spot problem. The HCDD and CoDA algorithms in the cluster head selection are conditional and relatively stable. By searching for the solution for minimizing the maximum distance between adjacent nodes and the maximum number of hops simultaneously, CoDA obtains the optimal path for cluster heads to deal with inter-cluster communication. Therefore, it has a better effect on energy consumption.

**Figure 12:**
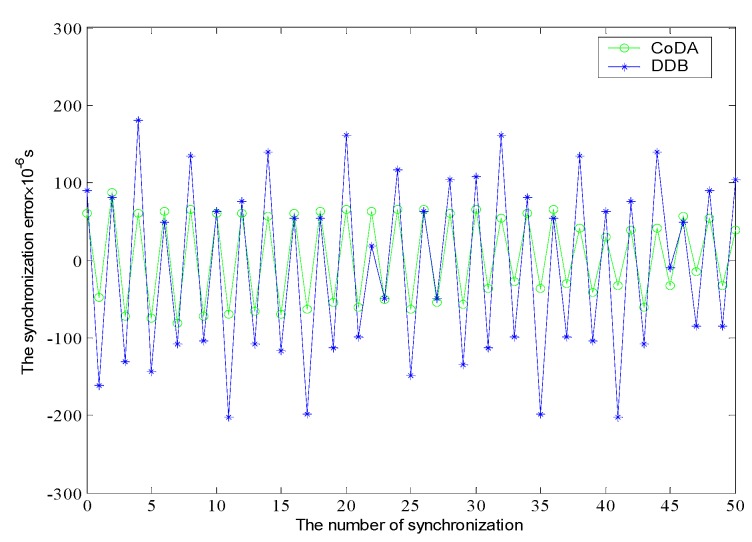
Comparison of synchronization error.

Finally, the synchronization error and overhead of the CoDA and DDB protocol are analyzed. The time derivation is set as sending node broadcasts a message until it being receives by the destination node. Figure 12 shows the synchronization error comparison. Each error variable is independent and follows the Gauss distribution. In the early stage of synchronization, the synchronization error of the two protocols is not quite different. With the time elapse, it can be observed that CoDA demonstrate much better than DDB in aspect of synchronization error, and the variation range of error value is stable and narrow.

**Figure 13:**
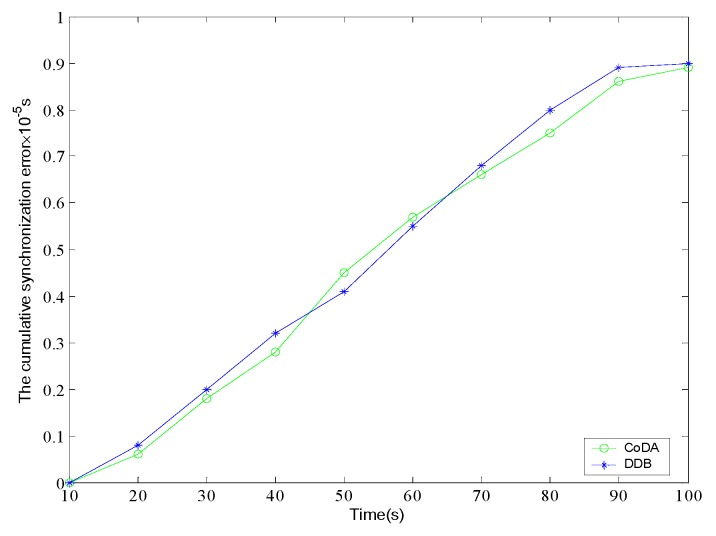
Comparison of the cumulative synchronization error.

Figure 13 shows the comparison of cumulative synchronization error of different protocols. In the experiment, the synchronization of 4 hops is set up, and the simulation time is limited to 120 s. In the initial stage, DDB can obtain relatively better precise synchronization than CoDA. Generally, the differences of cumulative synchronization error between DDB and CoDA are not significant. With the passage of time, the cumulative synchronization error demonstrates an increasing trend.

**Figure 14:**
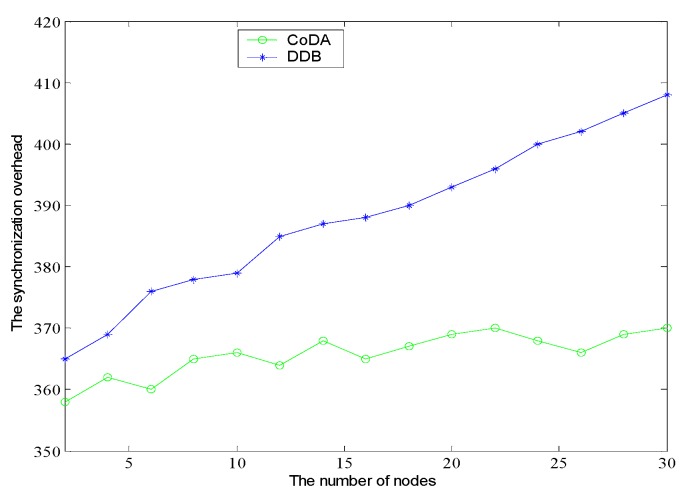
Comparison of the synchronization overhead.

As shown in Figure 14, the CoDA and DDB algorithms are compared to the synchronization overhead. Before the burst packet is sent, the RTS packet is 20 bytes, CTS is 14 bytes, the data frame is 2346 bytes. Besides, the reply message is a fixed length. Therefore, the effect of RTS/CTS on the convergence rate is very small. From the experimental results, it can be seen that the number of synchronous information packets of CoDA can be controlled at a lower level with the increase of the node. That is because the inter-cluster communication by multi-hop mechanism in CoDA can reduce a large number of overhead packets compared to DDB, which can reduce the power consumption of cluster heads. In addition, the number of synchronization messages is related to the number of nodes in the network. The number of nodes is more, the greater the synchronization overhead is. In general, the CoDA has the advantages of less accumulation error, high synchronization accuracy and good availability.

## 6. Conclusions

Topology control is a basic problem in wireless sensor networks, and it is also one of the important supporting technologies for large-scale applications. By means of reasonable topology control mechanism, the networks can work stably and effectively, thus extending their lifetime and reducing communication countermeasures. In this paper, a bio-level Voronoi diagram is proposed, which is a high energy efficiency data collection protocol that integrates topology control, MAC and routing. The research of this paper can provide a topology control mechanism and methods of network planning and dynamic maintenance. Also, it offer a powerful optimization mechanism and application example for research on variable, complex constraints and real-time requirements in large-scale and complex networks, therefore, our work has both a certain theoretical significance and practical application value.
